# A Sustainable Material for Sheep’s Cheese Wedges Stored under Different Atmosphere Conditions

**DOI:** 10.3390/foods13091423

**Published:** 2024-05-06

**Authors:** Ana Isabel Nájera, Maider Murua, Olaia Martínez, Marta Albisu, Luis Javier R. Barron

**Affiliations:** 1 Lactiker Research Group, Pharmacy and Food Science Department, Faculty of Pharmacy, Universidad del País Vasco/Euskal Herriko Unibertsitatea, 01006 Vitoria-Gasteiz, Spain; marta.albisu@ehu.eus (M.A.); luisjavier.rbarron@ehu.eus (L.J.R.B.); 2 Interal, 20100 Guipúzcoa, Spain; m.murua@interal.es; 3 Texture Analysis Laboratory, G3S Research Group, Pharmacy and Food Science Department, Faculty of Pharmacy, Universidad del País Vasco/Euskal Herriko Unibertsitatea, 01006 Vitoria-Gasteiz, Spain; olaia.martinez@ehu.eus

**Keywords:** packaging, recyclable material, cheese preservation, modified atmosphere, vacuum

## Abstract

This study is based on the need to improve packaging sustainability in the food industry. Its aim was to assess the performance of a recyclable plastic material for semi-hard sheep’s cheese wedges packaging as an alternative to conventional non-sustainable plastic materials. Four different packaging treatments (air, vacuum, and CO_2_/N_2_ gas mixtures 50/50 and 80/20% (*v*/*v*)) were studied. Changes in gas headspace composition, sensory properties, cheese gross composition, weight loss, pH, colour, and texture profile were investigated at 5 ± 1 °C storage for 56 days. The sensory analysis indicated that vacuum packaging scored the worst in paste appearance and holes, and air atmosphere the worst in flavour; it was concluded that cheeses were unfit from day 14–21 onwards. Air and vacuum packaging were responsible for most of the significant changes identified in the texture profile analysis, and most of these happened in the early stages of storage. The colour parameters a* and b* differentiated the air packaging from the rest of the conditions. As in previous studies using conventional plastic materials, modified atmosphere packaging, either CO_2_/N_2_ 50/50 or 80/20% (*v*/*v*), was the most effective preserving technique to ensure the quality of this type of cheese when comparing air and vacuum packaging treatments.

## 1. Introduction

The United Nations urge for a change of lifestyle and encourage effective action regarding climate change and against environmental pollution [1,2]. In 2021, more than 90% of the world’s plastic production is fossil based, while post-consumer recycled plastics only account for 8.3% [3]. In spite of that, plastic usage continues to grow due to great technological versatility [4]. This material is widely used for food packaging in Europe, as it is lightweight, reduces transport costs, and offers great design versatility, good barrier properties, chemical and thermal resistance, and appropriate containment properties, among others. Europeans generate about 30 kg of plastic packaging waste per person a year, and most of these are used only once [5]. In addition, around one third of all of the food produced worldwide is wasted unnecessarily [6,7].

In view of the above considerations, recyclable materials are one of the pillars promoted by the European Union (EU) for sustainable packaging and it is an expanding field [5,7]. Furthermore, these new alternative materials must also maintain their specific functions towards food, such as protecting, maintaining quality and safety, and extending shelf life [7,8]. It is necessary as well to assess the suitability of new and more sustainable materials for cheese storage as alternatives to current single-use packaging. Developing food-grade recycled materials implies a close control of the raw materials and their processing [9,10,11].

The most common polymeric barrier materials are etilen-vinil-alcohol (EVOH), polyvinylidene chloride (PVDC), and polyamide (PA), which can be combined in three- to seven-layer films to optimize their functionality, although multilayer films are difficult to recycle [12].

EVOH is widely used to improve the properties of bulk plastics [3], acting as a barrier to oxygen. In the presence of moisture, this material suffers the degradation of its barrier and mechanical properties. Therefore, it is commonly used in multilayer structures with moisture barrier materials, such as polyethylene (PE) or polypropylene (PP), to prevent its barrier properties. More than 5% EVOH within the total weight of the PE film affects the recyclability of the PE film [13]. Additionally, PE was designed to have one of the highest recycling potentials [14]. Moreover, PP is usually described as a material with better recyclability indexes [15,16]. PA is permeable to water vapor, but impermeable to oxygen and other gases, as well as being highly transparent. In flexible food packaging, the most common structures include complex laminates of PA film with PE film [16].

Idiazabal Protected Designation of Origin (PDO) cheese is developed in Northern Spain from Latxa sheep milk. Most of the farmers have their own flocks, with partial or extensive time spent grazing, which helps with the sustainability of the region [17,18]. Producers have great interest in decreasing the impact of this traditional foodstuff, while enhancing their commercial quality and shelf life. New packaging materials should be suitable to maintain atmosphere conditions, which are different depending on the characteristics of each cheese [8,19,20,21]. Vacuum and modified atmosphere packaging (MAP) have been widely used to prolong the shelf life of cheeses, combined with refrigerated temperature. Vacuum packaging offers protection against oxidative damage and inhibits aerobic bacteria, moulds, and yeasts. However, this technique is not suitable for some kinds of cheeses, as it may lead to undesirable changes [22,23]. MAP conditions might help to improve the appearance of the product, reduce microbial growth, and prevent chemical degradation, among others [24]. Both atmospheres help to avoid the use of antimicrobial additives, which may be positively perceived by consumers, and may contribute to extended shelf lives as well, which would lead to less cheese waste [8].

Current changes in lifestyle make consumers demand more portioned cheeses, in which alterations can occur faster [22,25]. In a previous study [23], the behaviour of PDO Idiazabal cheese wedges packaged in traditional non-recycled plastic was assessed (PA/PE 20/70 pouches of 90 µ thickness).

This study focuses on the current context of climate crisis, as well as on the consumer and sector demand for sustainability. For this reason, the aim of this study is to assess the performance and suitability of a recyclable plastic material for the packaging of semi-hard sheep’s cheese wedges under air, vacuum, and two different MAP conditions. Sensory and physicochemical parameters were addressed during the 56 days of refrigerated storage.

## 2. Materials and Methods

### 2.1. Properties of the Recyclable Plastic Material

A so-called Next flex mpox B 60 material was tested (Merkapack, Vitoria-Gasteiz, Spain). It is a multilayer co-extrusion film, composed of three materials as follows: 16 µ correspond to PP, 3 µ to EVOH, and 41 µ to PE. The three materials provided a total thickness of 60 µ. Permeability characteristics for oxygen were <3 cm^3^/m^2^ × bar × 24 h (75% r.h.) [26], for carbon dioxide <15 cm^3^/m^2^ × bar × 24 h (0% r.h.) (calculated), and for water vapour of <1 g/m^2^/24 h (calculated).

The recyclable plastic reduces the weight by 23%, and by 15 µm in thickness, and boasts 87% recyclability for packaging as compared to the standard material.

### 2.2. Cheese Samples

Forty raw sheep’s milk cheeses were used (Idiazabal PDO cheeses) after a three-month ripening time. They were supplied by a local dairy farm and produced separately in two groups within 3 days of each other (Batch A and Batch B).

Each cheese was cut into six wedges of approximately 180 g each, and 240 wedges were obtained. They were distributed into four groups of fifty-six wedges (28 were for both Batch A and B). The packaging environment was as follows: air, vacuum, 50% CO_2_/50% N_2_ (MAP1), and 80% CO_2_/20% N_2_ (MAP2). After being packaged in the material described in Section 2.1, cheeses were refrigerated at 5 ± 1 °C for 56 days. A MAP equipment Irimar model EVT-450/20 (Lesaka, Spain), food-grade gases from Carburos Metálicos-Grupo Air Products (Cornellá de Llobregat, Spain), and a Witt binary hand-operated gas mixer model MM-2K N_2_/CO_2_ (Witten, Germany) were used to package the cheese wedges. The conditions of the injection of gases and the heat-sealing of the packing machine were adapted to the characteristics of the material.

Samples were collected on day 0 before packaging, on day 14, and every week thereafter for sensory, colour, and physicochemical analysis and to assess the instrumental texture.

### 2.3. Package Headspace Gas Composition Analysis

The analysis of the gas concentration inside each individual bag was carried out using a Witt gas analyser (O_2_/CO_2_) model Oxybaby (Witten, Germany). Immediately after packaging in the laboratory, the pouches were subjected to a visual inspection of the sealing area to check for possible failures.

### 2.4. Cheese Sensory Analysis

Sensory evaluation was conducted with nine assessors (five men and four women) who have previously participated in other cheese studies [27,28] or packaged cheese trials [23], and six belonged to the PDO Idiazabal official sensory panel [29]. The assessors were trained in three 60 min sessions prior to the beginning of the study in order to harmonise the Idiazabal cheese ratings. Seven assessors participated in each session during the weekly assays. Sensory assessments were performed in a standardised tasting room in accordance with the ISO standards [30].

Sensory analysis was divided into two parts. A first part for the assessment of texture and flavour, and a second part for the appearance of any paste and holes in the cheese wedges. In each session, eight cheese samples were presented rind free and cut into parallelograms of 1.5 cm × 1.5 cm × 5 cm for texture and flavour. Cheese samples were tempered at 17 ± 2 °C in a wine cellar (La Sommelière, LS34A model, Barcelona, Spain) and randomly presented, after being coded with a three-digit number obtained from Fizz software 2.40 H (Biosystemes, Couternon, France). Granny Smith apples and low-mineralization water were used to remove any flavour interference between samples. Subsequently, the appearance of paste and holes was assessed using whole wedges for each conservation treatment. These latter samples were assigned a random three-digit number, different from the first part and different for each session. Analyses were performed in duplicate (Batch A and B).

Cheese samples were scored by the assessors on a 7-point discontinuous quality scale, where 1 is the null value, 4 is medium, and 7 is the maximum, depending on how close they are to the optimal characteristics of the cheese. In addition to the scales, the cards contain a section for observations and defects. Scores lower than 4 indicated that the cheeses presented defects, and assessors were asked to identify which defects were perceived [29].

### 2.5. Physicochemical Analyses

Cheese wedges were weighed before packaging and on each sampling day on an Adam balance (Milton Keynes, UK).

A Zeutec model 110-A100-1 (NIR) infrared spectral analyser 2.0 (Rendsburg, Germany) was employed to determine the percentages of protein, fat, and dry matter. The measurement was duplicated for the homogeneous fraction, obtained by grating a portion of each wedge after removing approximately 1 cm of the rind.

The pH was measured in triplicate at different points of the wedges by means of a pH-meter model GLP21+ with a Hach penetration electrode (Düsseldorf, Germany), and the sample temperature was simultaneously controlled with a Testo penetration thermometer model 104-IR (Barcelona, Spain).

### 2.6. Colour Analysis

The colour measurements were performed with a Chroma Meter CR-200 Minolta (Madrid, Spain), taking three measurements on one side of the cheese paste. The colour coordinates L*, a*, and b* were measured using the standard illuminant D65 at a visual angle of 10°. The equipment was calibrated using a standard white plate. The colour expression yellow index (Yi) (Yi = (142.86b*)/L*), proposed by Rohm and Jaros [31] and used by Favati et al. [25] on Provolone and on Parmigiano Reggiano cheese [32], was chosen. The yellowness index Zi (Zi = 100 (L* + 16/116) − (b*/200)3) used by del Caro et al. and some others [33,34] was also explored. Yi and Zi were used earlier in the preservation of the Idiazabal sheep cheese wedges [23].

### 2.7. Instrumental Texture Analysis

A TA.XTPlus (Stable Micro System, Surrey, UK) texture analyser was used, and samples for the texture analysis were obtained as previously explained by Albisu et al. [23] in 1.0 cm × 1.25 cm × 1.25 cm cubes.

Seven of these cubes were used for the uniaxial compression test at a controlled temperature (15 ± 2 °C). The assay conditions were established as follows: 50% compression and 1 mm/s crosshead speed. The probe was an aluminium cylinder with a 2.5 cm diameter. From the force (N)–time (s) curves, three texture parameters were obtained as follows:

Maximum load (N), corresponding to the highest force registered during the positive compression cycle.

Slope (N/s), obtained in the linear region of the positive compression phase, and Compression work (N·s), measured as the area under the curve of the whole positive compression phase (from the beginning to the point corresponding to the 50% of compression).

The eight remaining cubes were used for texture profile analysis or TPA [35,36], in which samples were subjected to two consecutive compression cycles at 15%. The following parameters were measured and analysed as described by Albisu et al. [23]: hardness, adherence, springiness, cohesiveness, chewiness, and resilience.

### 2.8. Data Treatment and Statistical Analysis

SPSS IBM Statistics software version 28.0 (New York, NY, USA) was used for statistical analysis (SPSS INC., Chicago, IL, USA). The Kruskal–Wallis H test was used to check for significant differences in the sensory parameters and instrumental texture between treatments and throughout the storage time. The two-way analysis of variance (ANOVA) was used to determine the significant differences in the headspace, physicochemical, and colour parameters of the different packaging treatments over the storage period, using the packaging treatment and storage time as fixed factors. Subsequently, Tukey’s test was applied to pairwise comparisons between cheeses packaged under different treatments and on each sampling day separately.

A stepwise discriminant analysis (SDA) was applied to sensory, physicochemical, instrumental colour, and texture parameters in order to classify cheese samples from the different packaging methods. Statistical significance was declared at *p* ≤ 0.05.

## 3. Results and Discussion

### 3.1. Headspace Gas Composition

The O_2_ and CO_2_ concentrations in the headspace of the packages were analysed, except for the vacuum-packed samples.

**Figure 1:**
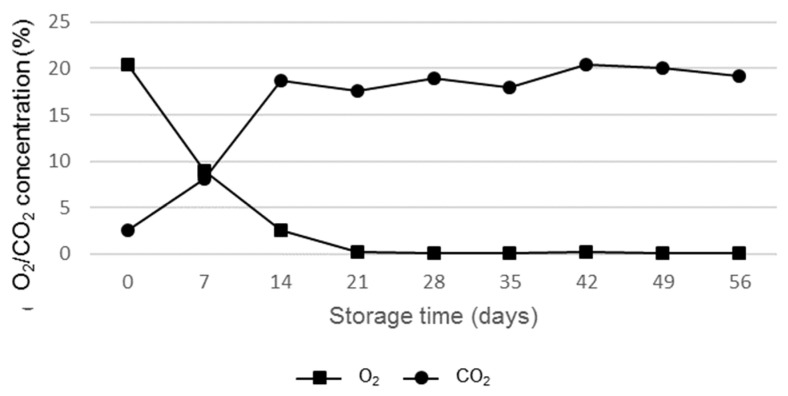
Evolution of the O_2_ and CO_2_ concentrations of cheese wedges stored for 56 days in the air-package treatment.

In the air treatment, the O_2_ concentration decreased eight times in the first fourteen storage days. From day 21 onwards, a significant difference was observed (*p* ≤ 0.01) as its presence was only 1.32% (Table 1; Figure 1). This may be due to the cheese microbiota, since the aerobic microorganisms could have been able to consume the O_2_ inside the package and convert it into CO_2_. It is less likely that this decrease in O_2_ happened due to gas permeation through the packaging material, given the low level of permeability (<3 cm^3^/m^2^ × bar × 24 h versus a value of ≤80 cm^3^/m^2^ × bar × 24 h of a conventional PA/PE 20/70 material).

A decrease in the O_2_ level from 19.98% to 0.16% was detected in previous experiences when dealing with Domiati cheese in cold air storage, despite using a very high oxygen barrier packaging film [37].

For both MAP1 and MAP2 treatments, the average O_2_ value was 0.12%, and it remained unchanged throughout the storage time. This residual amount of oxygen is indicative of the absence of failures in the container [38]. There were no significant differences in the O_2_ content among the MAP treatments, from 20 to 100% CO_2_-packaged cheeses (Samso and San Simón da Costa) [39,40], and the mean values were between 0.17 and 0.2%. Similar data (±0.4%) have recently been reported by Albisu et al. [23] in Idiazabal cheese wedges packaged in four different atmospheres, using a conventional non-recyclable material.

The air treatment presented a seven-fold increase in the first fourteen days and remained stable until the end of storage (Table 2; Figure 1). Considering the low CO_2_ permeability of this material (<15 cm^3^/m^2^ × bar × 24 h compared to a value of ≤174 for a conventional PA/PE 20/70 material), the progressive increase throughout the study could mainly be due to the microbial activities of the cheese [22].

In MAP conditions, unlike air packaging, there was a 17.62% average decrease in CO_2_ during storage. In both cases, after 14 days, MAP1 and MAP2 lost 10.23 and 13.21%, respectively. In the study with the PA/PE (20/70) non-recyclable material [23], a progressive decrease in the CO_2_ concentration was observed in the 20, 50, and 80% CO_2_ treatments, reaching 39% after eight weeks of storage. This decrease might be attributed to gas dissolution in the cheese matrix, its consumption by anaerobic microorganisms, or by CO_2_ loss through the barrier film [22,41]. In a similar study by Solomakos et al. [42] with sheep cheese packaged under 50/50% CO_2_/N_2_ conditions, after 10 storage days at 4 °C, the CO_2_ concentration decreased by 17.15% and remained stable thereafter.

In the first 21 storage days, the O_2_ was reduced by 98.68% and remained at a ratio of 0.27% until the end of the period. In a previous study using PA/PE (20/70) as the packaging material, this gas equilibrium was reached at a slower and more progressive rate in almost double the time (42 days) [23]. In addition to microbial growth, these results could be explained by the higher O_2_ and CO_2_ permeability of the PA/PE material, whereas the Next flex material used in this study presents a lower gas permeability. In low-permeability materials, a possible accumulation of water on the surface of the product could promote microbial growth [43,44]. For highly respiring products, a combination of low oxygen permeability and a high respiration rate easily leads to anaerobic conditions inside the package [45]. Therefore, film properties do influence the physicochemical cheese characteristics, and, consequently, the cheese microbiota. Related to this fact, Florit et al. [46] studied the effect of micro-perforation packaging material on the headspace atmosphere evolution.

### 3.2. Sensory Analysis

Concerning texture (Table 3), only a slight quality decrease (*p* ≤ 0.05) was observed across the two storage months for cheeses packaged in air and vacuum, but they were above the acceptance limit at all times (score out of 4). No significant differences in flavour (Table 3) were found for vacuum and MAPs, while those kept in air showed significant differences (*p* ≤ 0.001) at 14 days, and they were considered unfit from 21 days onwards. These samples displayed off-flavours, such as rancid, mouldy, or an undefined type. Although the oxygen present in the air packaging was low, it may have caused the mouldy and rancid off-flavours by favouring mould proliferation to a certain extend.

The paste appearance changed significantly in all packaging treatments across time, except for MAP2, which remained stable (Table 4). For air-packed cheeses, the only difference (*p* ≤ 0.05) was detected on day 56, where some assessors indicated slight greenish spots. This defect was caused by the proliferation of moulds and was related to the mouldy off-flavours. Vacuum-packaged cheeses had gradually decreasing scores, lowering 2.7 points from the beginning of the storage to the end. This decrease was the result of a plastic and shiny appearance, together with packaging marks and a non-homogeneous colour, which provided an anomalous appearance from day 14 onwards. This packaging treatment showed significantly lower scores (*p* ≤ 0.001) with respect to the rest of the treatments. The wedges packaged in MAP1 presented slight changes in the colour of the paste, which could be attributed to the cheese variability itself, and not to the packaging condition.

Regarding holes, significant differences (*p* ≤ 0.001) over time were only revealed for vacuum-packed cheeses. On day 14, holes started to occlude, and, at the end of the storage, cheeses presented cracks and sinkholes where holes were initially located. These defects were produced by the pressure necessarily exerted in vacuum packing, which produces anomalous colouring and holes.

In summary, vacuum packaging showed the worst results for paste appearance and holes. Air-packaged cheeses were the lowest rated for flavour and presented defects in the paste appearance at the end. Indeed, it has been reported that CO_2_ concentrations below 20% do not inhibit the growth of moulds [21,47]. Short storage times have been described for cheeses kept in air on account of mould growth [22,37,48]. MAP1 and MAP2 showed no significant changes for texture, flavour, and paste holes during the 56 storage days. In Parmigiano Reggiano cheese, increases in texture and flavour were detected, and, in MAP treatments, moisture and solubility were reduced, but no significant changes in flavour were recorded, except for vacuum-packed cheeses, in which bitterness increased.

Similar results were found in a previous study with Idiazabal PDO cheese using a non-recycled plastic material PA/PE (20/70), where the best-rated cheeses were packaged with 50/50 and 80/20% CO_2_/N_2_ [22]. Several authors pointed out that an atmosphere close to 50/50% CO_2_/N_2_ is the best for preserving the cheese flavour [32,48,49,50].

### 3.3. Physicochemical Analysis

Cheeses had mean values of 67.54 ± 1.11% for dry matter, 22.68 ± 0.59% for protein and 39.27 ± 0.90% for fat; the pH was 5.10 ± 0.05, and 0.11 ± 0.07% was quantified for the weight loss.

Dry matter and fat did not show significant differences (*p* ≤ 0.05), neither for packaging treatment or storage time, nor for the interactions between them. Protein showed some differences (*p* ≤ 0.05) due to packaging conditions and storage time, as well as for their interactions. In a previous study with Idiazabal PDO cheese using PA/PE (20/70) as the packaging material [23], none of the physicochemical parameters measured in wedges showed significant differences (*p* ≤ 0.05), neither for packaging treatment or conservation time, nor for the interactions between them. Kirkin et al. [51] and Solomakos et al. [42] did not observe significant changes in the composition of cheese samples throughout the study. The main protein content and total solids remained almost constant for six storage months in vacuum-packaged San Simon da Costa cheeses. The fat content remained constant, although fat migration was observed during storage [52].

pH levels showed some differences throughout the storage time, mainly between packing and 14 storage of days (an increase of 0.04 units for vacuum packaging and an average increase of 0.14 units for air and MAP treatments). The minimum and maximum values measured were 4.96 and 5.18, respectively. Regardless of the atmosphere composition, the pH value remained stable throughout the storage in a previous study concerning Idiazabal cheese [23]. Similar pH stability during refrigerated storage has also been reported in other MAP-packaged cheeses [51]. In vacuum-packaged cheeses, the pH remained constant for six storage months, with no significant differences in non-packaged samples [52]. It has been explained by lactose metabolization and lactic acid formation in the early stages of the processing, and by the low level of proteolysis.

Weight loss differences were observed for packaging treatments and conservation times (*p* ≤ 0.05) (Table 5). During storage, weight percentage losses remained constant for air and vacuum, whereas MAP1 and MAP2 increased their weight losses from day 42 onwards.

However, there were no statistically significant differences between the four treatments when comparing the percentage of total losses at the end of storage (mean value 0.13% ± 0.02), whereas losses of 0.15% were described for Provolone cheese [25]. After eight storage weeks of the Idiazabal cheese wedges, weight loss percentages of 0.39 ± 0.43% were reported without differences between the packaging treatments (air, vacuum, and MAP treatments) [23]. This is consistent with previous experiences where different gas mixtures did not significantly influence weight losses in MAP-packaged ripened cheeses [22,50,53]. The plastic material used prevented dehydration and weight loss [40].

### 3.4. Instrumental Colour Parameters

L* and Zi showed differences in terms of the storage time, a* and Yi for the packaging treatment, and b* for both (treatment and time) (Table 6). L*, a*, and Zi did not show any differences throughout the storage time for any of the treatments. Mean values ± standard deviation for L*, a* and b* colour parameters of the cheese wedges stored for 56 days at different treatments can be downloaded as Supplementary Materials (Table S1).

Air packaging (a* mean value −3.50) differentiated (*p* ≤ 0.01) from the rest of the treatments (a* mean value −2.35). This difference could be due to the presence of oxygen in the air treatment, which was able to initiate the colour change perceived via the instrumental measurement.

Throughout the storage time, b* remained stable only in air-packed cheeses (mean value of 13.44). In vacuum, MAP1, and MAP2, b* changed markedly during the first 14 days, decreasing from a mean value of 13.79 on day 0 to 11.60 on day 14, and remained almost stable until the end for all treatments.

Yi did not show significant differences throughout storage time in air- (mean value of 25.20) and MAP1-packed (mean value of 21.71) conditions. However, vacuum and MAP2 did show significant differences for Yi, which presented a 25.64 value on day 0 and a mean value of 21.45 on day 56 for both conditions.

In a previous study on Idiazabal cheese, a* and b* parameters were those with the highest discriminant weight between the preservation conditions. It should be highlighted that a* was able to statistically distinguish air-packaged cheeses from vacuum and MAP cheeses. In the same way, for b* and Yi, air and vacuum packaging treatments were similar, and different from MAP treatments, which were also like each other [23]. As in the present study, Yi and Zi did not seem to discriminate more than L*, a*, and b*.

In Provolone cheese packaged at different CO2 concentrations, no differences were detected in colour (Yi) [25], and Di Marzo et al. [54] suggested that both vacuum and protective atmospheres might stabilize the colour of cheese during storage. Kirkin et al. [51] did not observe significant changes for L*, while, in Parmiggiano Reggiano cheese packaged with 50%CO_2_/50%N_2,_ a decrease in L* was observed [32].

### 3.5. Instrumental Texture Analyses

**Figure 2:**
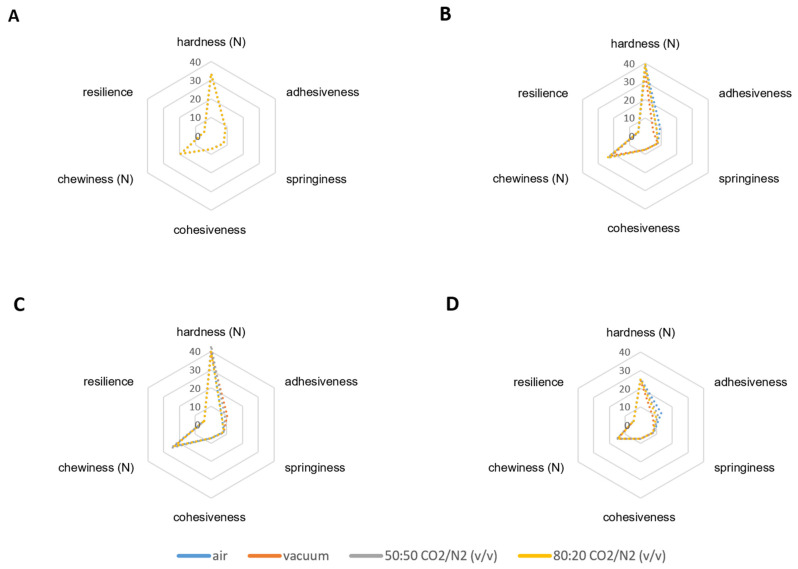
Texture profile based on the double compression test. Mean values for texture parameters are represented for day 0, 14, 35, and 56 from packing (**A**,**B**,**C**,**D** graphics, respectively), adapting the scale range for each case: hardness (×1), adhesiveness (−10^−1^), springiness (10^−1^), cohesiveness (10^−1^), resilience (10^−1^), chewiness (×1), and slope (×1).

In the storage time, there were generalized statistical differences (*p* < 0.05) in hardness, slope, chewiness, cohesiveness, and resilience for all atmospheres. Air samples remained stable in terms of cohesiveness and resilience. Figure 2 shows changes in the overall texture profile when comparing the initial sampling days with the last ones.

For all the treatments, the hardness decreased by 75% from the t0 values (33.413 N) to the end (*p* ≤ 0.001) after increasing to higher values in the intermediate weeks (38.267 N–40.677 N). Chewiness registered a 78% drop from t0 (19.337 N) to 56 days (*p* ≤ 0.001), with the middle values being (23.061 N–23.898 N). The adhesiveness increased significantly (*p* ≤ 0.001) by the end of the storage for MAP1 and air. The same tendency was identified in results from the uniaxial compression test, with the lowest values for the parameters measured in the last weeks, especially for the maximum load (Table 7). Uniaxial compression had an initial increasing behaviour in the three parameters measured, which generally registered the highest values in days 28 or 35. After that, values tended to decrease, nearing values of the initial days. This behaviour was also described in our previous contributions from Albisu et al. [23] when working with the same kind of cheese, but with a non-recyclable plastic packaging. Texture changes happened more slowly with the current recyclable material when compared to our previous work, as well as to some others, such as Atallah et al. [37] and Costa el al. [22], in which the maximum hardness and cohesiveness values were achieved 20–30 days after storage and then decreased, agreeing with gas mixture changes in the modified atmosphere. The current observation is probably due to the stabilization effect offered by MAPs with the CO_2_ content above 50% [23,49].

Data for the texture profile analysis (TPA) revealed few statistically significant differences (*p* ≤ 0.05) among the atmosphere conditions. Air and vacuum packaging were responsible for most of the significant changes identified, and the majority of these happened in the early stages of storage, after around 14 days of packaged refrigerated storage. At that time, the vacuum treatment had a significantly lower hardness (34.920 ± 7.46 N) when compared to other conditions. In the last day, vacuum samples showed significantly higher resilience values (0.464 ± 0.022), as compared to all of the other treatments (0.445–0.447), and it had the highest cohesiveness values (0.744 ± 0.02), which were statistically different when compared to air and MAP1, which were not statistically different and had a mean cohesiveness value ± SD 0.732 ± 0.025. It is usual to find collapsed packaging in vacuum-packed cheeses [55]. At 14 days of refrigerated storage, the treatment was statistically different in paste appearance and holes. It had the lowest mean values for cohesiveness and resilience, being 0.736 ± 0.218 and 0.436 ± 0.022, respectively. As suggested by sensory observations, a rapid growth of anaerobic bacteria and mould due to quick gas mixture changes could be responsible for these changes in the air-packed samples [22].

Focusing on uniaxial compression (Table 7), vacuum-packed samples displayed a behaviour which was slightly different to the others. The maximum load was the most outstanding parameter, as it showed a general tendency towards higher values in these samples, mostly from day 35 onwards. This tendency showed statistical significance (*p* ≤ 0.05) at day 42 (comparing to 50/50 CO_2_/N_2_ samples) and day 56, compared to both MAP1 and MAP2. The maximum load represents the highest force needed to cause the fractures registered during the compression test, thus, this should not be interpreted as hardness or firmness, as it would be the maximum peak force in TPA. The need for higher forces to fracture the samples, with the higher cohesiveness described for vacuum cheese samples in the double compression assay, is significant.

### 3.6. Discriminant Analysis

**Figure 3:**
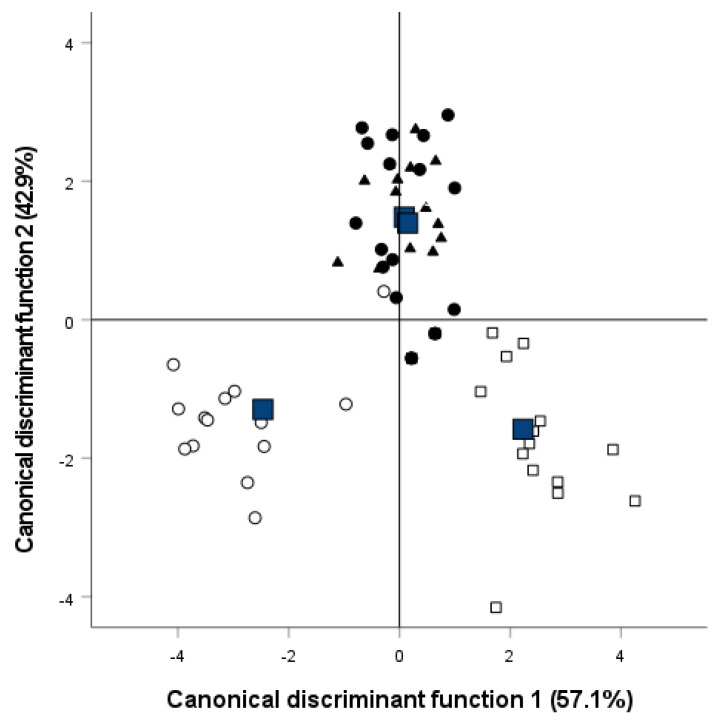
Graph for the two first canonical discriminant functions corresponding to the stepwise discriminant analyses of sensory, physicochemical, instrumental colour, and texture parameters of packaged cheese wedges stored for 56 days with different treatments. Air: □; vacuum: ○; 50% CO_2_ MAP: ▲; 80% CO_2_ MAP: ●.

Air-packaged samples were clearly differentiated from vacuum-packaged samples, and both types of packaging were also differentiated from MAPs (Figure 3). The results showed that 92.2% of the samples were correctly classified into their corresponding treatment group, taking 50% and 80% CO_2_ MAP as a single treatment group (Figure 3).

The cross-validation method used for sample classification reported that 93.8%, 87.5%, and 81.3% of the MAP, air-packaged, and vacuum-packaged samples were correctly assigned.

The discriminant variables with higher correlations with canonical functions in the structure matrix were paste appearance and holes, flavour, and yellowness (b*) and yellow index (Yi) colour parameters.

## 4. Conclusions

The headspace atmosphere changes observed during the storage of cheeses with the recyclable plastics material are coherent with those previously described with cheeses conserved in conventional plastic material. Given the low O_2_ and CO_2_ permeability of the current material, the equilibrium of both gases was reached faster.

Air-packaged samples were clearly differentiated from vacuum-packaged samples, and both types of packaging were also differentiated from MAPs, which did not differ from each other. Air and vacuum packaging were responsible for most of the significant changes identified in the texture and sensory profile analysis, which mostly occurred in the early stages of storage. Vacuum packaging scored the worst in terms of the paste appearance and holes, and air atmosphere the worst in flavour, with cheeses being considered unfit from day 14–21 onwards. The colour parameters a* and b* differentiated air packaging from the rest of the conditions. Consequently, the MAP conditions were the most suitable for cheese packaging, providing results similar to those described with conventional plastic materials.

These results show the possibility of using recyclable plastic material for semi-hard cheese packaging, ensuring the quality of this kind of cheese, while reducing plastic waste and improving the sustainability of the food industry. It is essential to carry out more studies to determine the suitability of other sustainable materials (biodegradable) for food storage, and encourage the food industry to adopt other food packaging products.

## Figures and Tables

**Table 1:** Mean, standard deviation, and significance level of ANOVA for O_2_ concentration (%) in the headspace of packaged cheese wedges stored for 56 days with different treatments.

Day	Air	MAP1	MAP2
0	20.46 ± 0.14 ^a^	0.32 ± 0.18 ^a^	0.18 ± 0.04 ^a^
14	2.54 ± 0.32 ^b^	0.17 ± 0.15 ^a^	0.05 ± 0.06 ^a^
21	0.27 ± 0.00 ^c^	0.05 ± 0.02 ^a^	0.04 ± 0.01 ^a^
28	0.13 ± 0.13 ^c^	0.13 ± 0.13 ^a^	0.14 ± 0.01 ^a^
35	0.10 ± 0.13 ^c^	0.06 ± 0.05 ^a^	0.10 ± 0.12 ^a^
42	0.15 ± 0.08 ^c^	0.08 ± 0.14 ^a^	0.05 ± 0.07 ^a^
49	0.09 ± 0.12 ^c^	0.03 ± 0.01 ^a^	0.06 ± 0.02 ^a^
56	0.03 ± 0.03 ^c^	0.11 ± 0.04 ^a^	0.26 ± 0.19 ^a^
	**	NS	NS

MAP1: 50/50 CO_2_/N_2_ (*v*/*v*); MAP2: 80/20 CO_2_/N_2_ (*v*/*v*). Different letters in the same column indicate significant differences during storage for each packaging treatment. ** *p* ≤ 0.01; NS: non-significant differences.

**Table 2:** Mean, standard deviation, and significance level of ANOVA for CO_2_ concentration (%) in the headspace of packaged cheese wedges stored for 56 days with different treatments.

Day	Air	MAP1	MAP2
0	2.60 ± 0.28 ^b^	54.25 ± 0.99 ^a^	86.80 ± 0.99 ^a^
14	18.68 ± 0.81 ^a^	48.70 ± 0.35 ^b^	75.33 ± 1.03 ^b^
21	17.65 ± 1.91 ^a^	45.75 ± 1.34 ^b c^	75.75 ± 0.78 ^b^
28	18.90 ± 0.71 ^a^	43.80 ± 0.85 ^c^	71.70 ± 2.12 ^b^
35	17.95 ± 0.21 ^a^	43.55 ± 1.06 ^c^	71.25 ± 0.35 ^b^
42	20.45 ± 1.34 ^a^	46.10 ± 0.99 ^b c^	74.65 ± 0.70 ^b^
49	20.10 ± 0.14 ^a^	46.50 ± 0.42 ^b c^	73.40 ± 1.70 ^b^
56	19.15 ± 0.92 ^a^	44.70 ± 1.41 ^c^	71.50 ± 0.85 ^b^
	**	**	**

MAP1: 50/50 CO_2_/N_2_ (*v*/*v*); MAP2: 80/20 CO_2_/N_2_ (*v*/*v*). Different letters in the same column indicate significant differences during storage for each packaging treatment. ** *p* ≤ 0.01.

**Table 3:** Mean values ± standard deviation for the sensory texture and flavour of packaged cheese wedges stored for eight weeks with different treatments. Level of significance for Kruskal–Wallis H test is shown to indicate differences between treatments and storage time.

Texture					
Day	Air	Vacuum	MAP1	MAP2	
0	5.21 ± 0.43 ^1^	5.21 ± 0.43 ^1^	5.21 ± 0.43	5.21 ± 0.43	NS
14	4.79 ± 0.70 ^12^	4.57 ± 0.65 ^12^	4.86 ± 0.53	4.86 ± 0,66	NS
21	5.21 ± 0.58 ^1^	4.79 ± 0.70 ^12^	4.86 ± 0.77	4.79 ± 0.80	NS
28	4.29 ± 0.73 ^12^	4.29 ± 0.47 ^2^	4.93 ± 0.62	4.86 ± 0.77	NS
35	4.79 ± 0.70 ^12^	4.71 ± 0.73 ^12^	5.21 ± 0.58	5.00 ± 0.68	NS
42	4.71 ± 0.73 ^12^	4.93 ± 0.73 ^12^	4.71 ± 0.73	4.79 ± 0.80	NS
49	4.86 ± 0.36 ^12^	4.86 ± 0.86 ^12^	5.07 ± 0.62	4.79 ± 0.70	NS
56	4.50 ± 0.52 ^2^	4.79 ± 0.80 ^12^	4.50 ± 0.85	4.57 ± 0.76	NS
	*	*	NS	NS	
**F** **lavour**					
0	5.14 ± 0.53 ^1^	5.14 ± 0.53	5.14 ± 0.53	5.14 ± 0.53	NS
14	4.29 ± 0.45 ^12^	4.50 ± 0.65	5.00 ± 0.78	4.79 ± 0.68	NS
21	3.71 ± 0.83 ^b 234^	5.14 ± 0.77 ^a^	4.71 ± 0.83 ^a^	4.79 ± 0.58 ^a^	**
28	3.86 ± 0.96 ^b 23^	4.64 ± 0.74 ^ab^	4.93 ± 0.73 ^a^	4.79 ± 0.80 ^ab^	**
35	3.29 ± 0.73 ^b 234^	4.71 ± 0.47 ^a^	4.57 ± 0.51 ^a^	5.14 ± 0.66 ^a^	***
42	2.86 ± 0.36 ^b 34^	5.14 ± 0.86 ^a^	4.79 ± 0.80 ^a^	4.93 ± 0.62 ^a^	***
49	3.36 ± 0.84 ^b 234^	4.93 ± 0.83 ^a^	5.14 ± 0.53 ^a^	4.71 ± 0.73 ^a^	***
56	2.64 ± 0.64 ^b 34^	5.00 ± 0.78 ^a^	4.50 ± 0.94 ^a^	4.43 ± 0.85 ^a^	***
	***	NS	NS	NS	

MAP1: 50/50 CO_2_/N_2_ (*v*/*v*); MAP2: 80/20 CO_2_/N_2_ (*v*/*v*). Different letters in the same row indicate significant differences between the different packaging conditions on that day. Different numbers in the same column indicate significant differences during storage for each packaging condition. * *p* ≤ 0.05; ** *p* ≤ 0.01; *** *p* ≤ 0.001; NS: non-significant differences.

**Table 4:** Mean values ± standard deviation for sensory paste appearance and holes of packaged cheese wedges stored for eight weeks at different treatments. Level of significance of Kruskal–Wallis H test is shown to indicate differences between treatments and storage time.

Paste Appearance					
Day	Air	Vacuum	MAP1	MAP2	
0	5.36 ± 0.50 ^1^	5.36 ± 0.50 ^1^	5.36 ± 0.50 ^12^	5.36 ± 0.50	NS
14	5.71 ± 0.45 ^a 1^	3.57 ± 0.65 ^b 12^	5.64 ± 0.50 ^a 1^	5.50 ± 0.85 ^a^	***
21	5.14 ± 0.86 ^a 12^	3.07 ± 0.47 ^b 23^	5.00 ± 0.88 ^a 12^	5.00 ± 0.88 ^a^	***
28	5.21 ^a^ ± 0.80 ^a 12^	2.93 ± 0.73 ^b 23^	5.50 ± 0.76 ^a 1^	5.50 ± 0.76 ^a^	***
35	5.00 ± 0.55 ^a 12^	2.71 ± 0.47 ^b 23^	5.14 ± 0.66 ^a 12^	5.00 ± 0.68 ^a^	***
42	5.50 ± 0.52 ^a 1^	2.57 ± 0.65 ^b 23^	5.36 ± 0.63 ^a 12^	5.43 ± 0.5 ^a^	***
49	4.93 ± 0.47 ^a 12^	2.43 ± 0.76 ^b 3^	5.21 ± 0.89 ^a 12^	5.43 ± 0.65 ^a^	***
56	4.14 ± 0.73 ^a 2^	2.36 ± 0.74 ^b 3^	4.50 ± 0.65 ^a 2^	4.79 ± 0.58 ^a^	***
	*	***	*	NS	
**P** **aste holes**					
0	5.21 ± 0.43	5.21 ± 0.43	5.21 ± 0.43	5.21 ± 0.43	NS
14	5.21 ± 0.86 ^a^	3.64 ± 0.63 ^b 2^	5.29 ± 0.61 ^a^	4.86 ± 0.77 ^a^	**
21	5.07 ± 0.83 ^a^	3.29 ± 0.73 ^b 2^	5.00 ± 0.78 ^a^	5.14 ± 0.77 ^a^	***
28	4.71 ± 0.91 ^a^	2.79 ± 0.43 ^b 23^	5.00 ± 0.88 ^a^	4.43 ± 0.94 ^a^	***
35	5.14 ± 0.36 ^a^	2.71 ± 0.47 ^b 23^	5.07 ± 0.83 ^a^	5.07 ± 0.27 ^a^	***
42	4.79 ± 0.58 ^a^	2.86 ± 0.36 ^b 23^	5.07 ± 0.73 ^a^	5.29 ± 0.73 ^a^	***
49	5.29 ± 0.61 ^a^	2.71 ± 0.47 ^b 23^	5.00 ± 0.68 ^a^	5.21 ± 0.70 ^a^	***
56	4.86 ± 0.73 ^a^	2.29 ± 0.61 ^b 3^	5.07 ± 0.92 ^a^	4.93 ± 0.92 ^a^	***
	NS	***	NS	NS	

MAP1: 50/50 CO_2_/N_2_ (*v*/*v*); MAP2: 80/20 CO_2_/N_2_ (*v*/*v*). Different letters in the same row indicate significant differences between the different packaging conditions on that day. Different numbers in the same column indicate significant differences during storage for each packaging condition. * *p* ≤ 0.05; ** *p* ≤ 0.01; *** *p* ≤ 0.001; NS: non-significant differences.

**Table 5:** Mean values ± standard deviation for weight loss (%) of the cheese wedges stored for 56 days with different treatments. Significance level of ANOVA is shown for differences in the storage time.

Day	Air	Vacuum	MAP1	MAP2
14	0.07 ± 0.04 ^a^	0.09 ± 0.02 ^a^	0.06 ± 0.04 ^b^	0.03 ± 0.04 ^bc^
21	0.15 ± 0.04 ^a^	0.12 ± 0.02 ^a^	0.08 ± 0.02 ^b^	0.01 ± 0.01 ^c^
28	0.13 ± 0.03 ^a^	0.15 ± 0.04 ^a^	0.07 ± 0.03 ^b^	0.09 ± 0.06 ^abc^
35	0.11 ± 0.04 ^a^	0.08 ± 0.03 ^a^	0.06 ± 0.06 ^b^	0.02 ± 0.02 ^c^
42	0.21 ± 0.04 ^a^	0.26 ± 0.16 ^a^	0.22 ± 0.01 ^a^	0.13 ± 0.01 ^a^
49	0.14 ± 0.00 ^a^	0.15 ± 0.00 ^a^	0.15 ± 0.03 ^ab^	0.13 ± 0.04 ^a^
56	0.13 ± 0.03 ^a^	0.14 ± 0.00 ^a^	0.13 ± 0.04 ^ab^	0.11 ± 0.03 ^ab^
	NS	NS	*	*

MAP1: 50/50 CO_2_/N_2_ (*v*/*v*); MAP2: 80/20 CO_2_/N_2_ (*v*/*v*). Different letters in the same column indicate significant differences (*p* ≤ 0.05) during storage for each packaging treatment. * *p* ≤ 0.05; NS: non-significant differences.

**Table 6:** Mean values ± standard deviation for overall colour parameters L*, a*, b*, Yi, and Zi. Significance levels are shown for the ANOVA test regarding packaging treatment, storage time, and interactions between them.

	L*	a*	b*	Yi	Zi
**Packaging treatment**	NS	**	**	**	NS
**Storage time**	**	NS	**	NS	**
**Interaction**	NS	NS	NS	NS	NS
**x ± SD**	75.80 ± 1.49	−2.58 ± 0.78	11.83 ± 1.04	22.30 ± 1.89	39.29 ± 2.02

L*: lightness; a*: redness; b*: yellowness; yellow index (Yi): (142.86b*)/L*; yellowness index (Zi): 100(L* + 16/116) − (b*/200)^3^. ** *p* ≤ 0.01; NS = not significant differences.

**Table 7:** Mean values ± standard deviation for texture parameters obtained in the compression test for cheese wedges stored for eight weeks with different treatments. Level of significance of Kruskal–Wallis H test is shown to indicate differences between treatments and storage time.

	Day	Air	Vacuum	MAP1	MAP2	
**ML (N)**	0	41.910 ± 10.750 ^2^	41.916 ± 10.750 ^2^	41.916 ± 10.750 ^2^	41.916 ± 10.750 ^12^	NS
	14	46.776 ± 13.435 ^12^	50.190 ± 14.978 ^12^	49.175 ± 11.822 ^1^	47.937 ± 13.357 ^1^	NS
	21	42.696 ± 8.273 ^12^	42.394 ± 8.165 ^12^	41.894 ± 8.120 ^12^	39.592 ± 6.745 ^12^	NS
	28	48.447 ± 9.765 ^1^	47.217 ± 8.364 ^1^	47.716 ± 8.588 ^1^	47.852 ± 12.959 ^12^	NS
	35	48.853 ± 7.190 ^1^	50.232 ± 7.846 ^1^	47.851 ± 8.851 ^1^	47.001 ± 9.676 ^12^	NS
	42	37.464 ± 7.997 ^23 ab^	40.183 ± 9.208 ^123 a^	34.863 ± 6.028 ^23 b^	37.542 ± 7.602 ^2 ab^	*
	49	39.664 ± 7.997 ^2^	40.829 ± 5.755 ^2^	39.673 ± 7.211 ^2^	40.740 ± 6.308 ^12^	NS
	56	31.627 ± 6.688 ^3 ab^	34.444 ± 5.379 ^3 a^	31.481 ± 6.074 ^3 b^	31.285 ± 5.077 ^3 b^	*
		**	**	**	**	
**CW (N·s)**	0	248.748 ± 66.633 ^2^	248.748 ± 66.633 ^2^	248.748 ± 66.633 ^2^	248.748 ± 66.633 ^2^	NS
	14	299.481 ± 97.410 ^12^	317.810 ± 120.620 ^1^	302.133 ± 71.926 ^1^	291.413 ± 71.020 ^12^	NS
	21	271.601 ± 74.114 ^12^	263.631 ± 48.425 ^12^	268.384 ± 63.489 ^2^	250.631 ± 43.982 ^12^	NS
	28	208.583 ± 69.798 ^3 b^	230.622 ± 70.414 ^3 ab^	239.357 ± 61.118 ^2 ab^	255.805 ± 66.305 ^2 a^	*
	35	306.395 ± 82.274 ^1^	312.260 ± 54.760 ^1^	301.932 ± 68.075 ^1^	294.106 ± 61.663 ^1^	NS
	42	235.151 ± 60.292 ^2^	244.203 ± 54.038 ^2^	218.589 ± 43.724 ^23^	235.001 ± 60.746 ^23^	NS
	49	238.807 ± 54.916 ^2^	237.108 ± 43.323 ^234^	251.507 ± 51.229 ^2^	256.664 ± 55.653 ^2^	NS
	56	185.041 ± 38.145 ^3^	188.275 ± 43.258 ^4^	180.435 ± 44.191 ^3^	190.605 ± 34.233 ^3^	NS
		**	**	**	**	
**Slope (N/s)**	0	16.101 ± 5.972 ^3^	16.101 ± 5.972 ^3^	16.101 ± 5.972 ^3^	16.101 ± 5.972 ^23^	NS
	14	17.417 ± 10.276 ^3^	16.681 ± 10.618 ^3^	18.994 ± 6.514 ^3^	16.438 ± 8.608 ^23^	NS
	21	18.407 ± 6.567 ^3^	19.613 ± 6.135 ^2^	19.694 ± 5.1122 ^3^	18.852 ± 5.159 ^12^	NS
	28	30.036 ± 8.935 ^1 a^	21.535 ± 12.441 ^12 b^	26.933 ± 13.415 ^1 ab^	26.061 ± 12.950 ^1 ab^	*
	35	23.882 ± 7.941 ^12^	23.651 ± 8.320 ^1^	22.981 ± 8.024 ^12^	20.459 ± 8.357 ^1^	NS
	42	14.852 ± 7.709 ^3^	17.120 ± 5.902 ^23^	15.693 ± 7.442 ^3^	16.552 ± 4.965 ^23^	NS
	49	19.681 ± 5.342 ^23^	19.054 ± 6.298 ^23^	18.502 ± 6.940 ^3^	21.135 ± 5.493 ^1^	NS
	56	15.514 ± 5.280 ^3 ab^	17.552 ± 4.521 ^23 a^	17.051 ± 3.761 ^3 a^	14.179 ± 4.274 ^3 b^	*
		**	**	**	**	

MAP1: 50/50 CO_2_/N_2_ (*v*/*v*); MAP2: 80/20 CO_2_/N_2_ (*v*/*v*). ML: Maximum load; CW: compression work. Different letters in the same row indicate significant differences between the different packaging conditions on that day. Different numbers in the same column indicate significant differences during storage for each packaging condition. * *p* ≤ 0.05; ** *p* ≤ 0.01; NS: non-significant differences.

## Data Availability

The original contributions presented in the study are included in the article/Supplementary Materials, further inquiries can be directed to the corresponding author.

## Supplementary Materials

The following supporting information can be downloaded at: https://www.mdpi.com/article/10.3390/foods13091423/s1, Table S1. Mean values ± standard deviation for L*, a* and b* colour parameters of the cheese wedges stored for 56 days at different treatments. Significance level of ANOVA is shown for differences in the storage time.
