# Supplementary material for: A Sustainable Material for Sheep’s Cheese Wedges Stored under Different Atmosphere Conditions
Source: Foods. 2024 May 6;13(9):1423. doi: 10.3390/foods13091423 (PMC11083857; doi:10.3390/foods13091423)
Supplement: Supplementary file 1 [file foods-13-01423-s001.zip › foods-2960411-supplementary.pdf]

Supplementary file

Table S1. Mean values ± standard deviation for L*, a* and b* colour parameters of the cheese wedges stored for 56 days at different treatments. Significance level of ANOVA is shown for differences in the storage time.

	Day	Air	Vacuum	MAP1	MAP2
**L***	0	76.92±2.51[a]	76.92±2.51[a]	76.92±2.51[a]	76.92±2.51[a]
	14	77.16±2.00[a]	77.13±0.80[a]	74.99±0.86[a]	75.08±0.64[a]
	21	73.99±0.99[a]	75.39±1.42 [a]	75.79±1.44[a]	75.25±0.38[a]
	28	77.96±0.92[a]	76.58±1.61[a]	77.39±1.46[a]	77.16±1.12[a]
	35	77.62±0.95[a]	77.46±1.02[a]	76.95±0.95[a]	76.86±0.51[a]
	42	74.59±0.41[a]	74.29±1.33[a]	75.59±0.45[a]	73.95±0.62[a]
	49	76.11±1.37[a]	74.55±0.15[a]	75.60±0.46[a]	75.92±1.50[a]
	56	75.37±1.83[a]	74.04±2.22[a]	74.68±2.10[a]	75.04±0.73[a]
		NS	NS	NS	NS
**a***	0	-3.04±0.79[a]	-3.04±0.79[a]	-3.04±0.79[a]	-3.04±0.79[a]
	14	-3.79±0.49[a]	-2.02±0.72[a]	-2.20±0.52[a]	-2.46±0.28[a]
	21	-3.81±0.05[a]	-2.15±0.52 [a]	-2.15±0.44[a]	-2.42±0.51[a]
	28	-3.58±0.47[a]	-2.17±0.52[a]	-2.14±0.73[a]	-2.39±0.38[a]
	35	-3.41±0.23[a]	-2.19±0.17[a]	-2.15±0.98[a]	-2.21±0.78[a]
	42	-3.50±0.29[a]	-2.11±0.83[a]	-3.00±1.97[a]	-2.26±0.52[a]
	49	-3.25±0.49[a]	-1.85±0.53[a]	-2.04±0.85[a]	-2.31±0.54[a]
	56	-3.70±0.04[a]	-2.36±0.59[a]	-2.24±0.77[a]	-2.57±0.64[a]
		NS	NS	NS	NS
**b***	0	13.79±0.31[a]	13.79±0.31[a]	13.79±0.31[a]	13.79±0.31[a]
	14	13.65±0.29[a]	11.79±0.03[bc]	11.30±0.59[ab]	11.70±0.04[b]
	21	13.23±0.13[a]	11.40±0.33[bcd]	11.15±0.56[ab]	11.20±0.76[b]
	28	13.46±0.13[a]	11.46±0.09[bcd]	11.52±0.45[ab]	11.99±0.10[b]
	35	13.56±0.23[a]	12.09±0.35[b]	11.53±0.70[ab]	11.64±0.30[b]
	42	13.34±0.23[a]	10.75±0.37[d]	11.76±1.50[ab]	10.96±0.12[b]
	49	13.28±0.18[a]	10.62±0.10[d]	10.70±0.80[b]	11.02±0.22[b]
	56	13.22±0.34[a]	11.10±0.06[cd]	10.74±0.36[b]	11.28±0.30[b]
		NS	**	*	**

L*: lightness; a*: redness; b*: yellowness; MAP1: 50/50 CO_2/N_2 (v/v); MAP2: 80/20 CO_2/N_2 (v/v). Different letters in the same column indicate significant differences during storage for each packaging treatment. **p ≤ 0.01; * *p* ≤ 0.05; NS: non significant differences
